# Editorial: Prevention and treatment of depression and subjective cognitive decline in late life: the role of lifestyles

**DOI:** 10.3389/fpsyt.2023.1338088

**Published:** 2023-11-28

**Authors:** Wei Liang, Yanping Duan, Sonia Lippke, Yi-Qun Gan, Joseph Tak-Fai Lau

**Affiliations:** ^1^School of Physical Education, Shenzhen University, Shenzhen, China; ^2^Deparment of Sport, Physical Education and Health, Hong Kong Baptist University, Kowloon, Hong Kong SAR, China; ^3^Constructor University Bremen, Bremen, Germany; ^4^School of Psychological and Cognitive Sciences, Peking University, Beijing, China; ^5^Center for Health Behaviors Research, The Chinese University of Hong Kong, Shatin, Hong Kong SAR, China

**Keywords:** mental health, cognition, psychosocial factors, lifestyle, older adults, SCD, non-pharmacological approach, COVID-19

Subjective cognitive decline (SCD) and depression are common health issues affecting the elderly population. These conditions significantly impair individuals' daily functioning, placing a considerable burden on families and society (1, 2). The COVID-19 pandemic has exacerbated the prevalence of SCD and depression, particularly among older adults (3–5). Consequently, effective prevention and treatment of SCD and depression have become critical public health and medical imperatives. With the aim of shedding light on the effects and underlying mechanisms of non-pharmacological strategies for preventing and managing SCD and depression, our Research Topic entitled “Prevention and Treatment of Depression and Subjective Cognitive Decline in Late Life: The Role of Lifestyles” aims to explore the role of individuals' psychophysiological characteristics and lifestyle behaviors on these conditions. The Research Topic features six new papers that examine the intricate relationship between diverse influential factors and cognition or depression in older adults.

Two studies focused on the correlates of depressive symptoms in older adults. Park et al. conducted a community-based cohort study in Korea to investigate gender differences in the association between leisure-time physical activity (PA), resistance training (RT), and incident depression. Out of 3,967 participants who were not depressed at baseline, 432 (10.89%) developed depression at the 4-year follow-up. The results revealed that, for women, engaging in moderate-intensity leisure-time PA for 150–299 min/week and more than 300 min/week was associated with a risk reduction of 38 and 44% (both *p* < 0.05), respectively, compared to those who did not engage in PA. However, no significant association between PA engagement and decreased risk of depression was found in men, and RT did not significantly impact depression in either the low-PA or high-PA group (all *p* > 0.05). Wang et al. utilized cross-sectional data from the China Health and Retirement Longitudinal Study 2011 (CHARLS2011) to explore the correlation between 13 cardiometabolic factors and depression among a sample of 8,942 mid-aged and older adults in China. The study demonstrated that depression was prevalent in 41.1% of mid-aged and older men and 55.1% of women. The results also indicated that body mass index (BMI), lipid accumulation product, triglyceride glucose (TyG) and BMI correlation index (TyG-BMI), and triglyceride glucose and waist circumference (WC) correlation index (TyG-WC) were associated with depressive symptoms in both males and females (all *p* < 0.05). The waist-height ration, body roundness index, WC, and Chinese visceral adiposity index were associated with depressive symptoms only in men (all *p* < 0.05). Other cardiometabolic factors, including visceral adiposity index, a body shape index, conicity index, and TyG index were not significant correlates of depressive symptoms in the study sample (all *p* < 0.05).

Smith et al. utilized longitudinal data from the Program to Encourage Active, Rewarding Lives (PEARLS) to evaluate the program's effectiveness in reducing depressive symptoms among 1,155 community-dwelling older adults in the United States. In this study, 25.3 and 10.5% of participants reported experiencing moderately severe and severe depression, respectively, at the outset. The results indicated a significant decline in depressive symptoms over time (*p* < 0.05), suggesting promising benefits of the program. Additionally, the study highlighted a noteworthy distinction in the remission of depressive symptoms among individuals with varying levels of depression severity. Roskoschinski et al. conducted a cross-sectional study to investigate the relationship between loneliness, depression, self-efficacy and social support among 135 German patients with multimorbidity. In addition, it was tested whether there was difference in depressive symptoms between multimorbid patients who survived COVID-19 infection and those who were not infected previously, which was not the case (*p* > 0.05). The results revealed a positive correlation between loneliness and depression (*r* = 0.419, *p* < 0.001), while negative correlations were found between depression and self-efficacy, as well as social support (*r* = −0.577 to −0.388, *p* < 0.001). Additionally, this study identified the mediating role of self-efficacy in the relationship between loneliness and depression (indirect effect β = 0.111, *p* < 0.001), however, no support was found for the moderating role of social support (*p* < 0.05).

The primary focus of the remaining two studies was to examine the mediating effect of depressive symptoms on the relationship between cognitive function and its correlates among older adults. Hou et al. utilized cross-sectional data from the CHARLS2018 study to examine the correlation between cognitive function and caring for grandchildren based on living arrangements among 5,490 Chinese mid-aged and older adults. Moreover, the authors explored the potential mediating role of social activities and depressive symptoms in this association. The findings indicated a significant correlation between cognitive function and the act of caring for grandchildren, depending on participants' cohabitation status with a spouse (i.e., *B*_living with a spouse_ = 0.829, *p* < 0.001; *B*_not living with a spouse_ = −0.545, *p* < 0.05). Furthermore, both social activities and depressive symptoms significantly mediated the relationship between caring for grandchildren and cognitive function (β_indirect effect for social activities_ = 0.107, *p* < 0.001; β_indirect effect for depressive symptoms_ = 0.105, *p* < 0.001). Similarly, Hong et al. examined the mediating role of depressive symptoms in the association between digital literacy and cognitive function among 7,988 Korean older adults. The results revealed a significant correlation between four types of digital literacy (i.e., communication, information, media, and online transaction literacy) and both depressive symptoms (β = −0.702 to −0.123, *p* < 0.05) and cognitive function (β = 1.436 to 2.217, *p* < 0.001). A significant mediating effect of depressive symptoms on the relationship between media and online transaction literacy and cognitive function was identified in this study (all *p* < 0.001).

In conclusion, the papers compiled in this Research Topic offer valuable insights into the diverse correlates and underlying mechanisms of cognition and depression among older adults, demonstrating the importance of factors e.g., time and BMI, PA, and loneliness, caring for grandchildren and digital literacy. While some of these factors can directly be addressed in interventions, others need to be considered and addressed indirectly e.g., BMI by PA and loneliness by social activities. Especially loneliness might be an early indicator of social exclusion and feeling overwhelmed, which might serve as a warning factor (6). Especially with the finding that those older people who live with a spouse seem to experience a protecting potential for SCD, while the ones not living with a spouse are more at risk for too many demands and too few resources like social support, this harbors potential for better understanding, theory refinement and policy building.

Future research should continue to focus on the prevention and treatment of depression and SCD among older adults. Specifically, there is a need for more population-based surveillances, more evaluation of non-pharmacological programs (e.g., PA, cognitive training, social activities, and nutrition) using rigorous empirical designs (e.g., randomized controlled trials), and an investigation of the biological and psychosocial mechanisms of these conditions. Lastly, interventions addressing the simultaneous occurrence of depression and SCD among the elderly need to be developed, utilizing telehealth and other technology-based alternatives to traditional treatment methods i.e., hybrid ones improving social competences and wellbeing even in face of multi-morbidity.

## Author contributions

WL: Conceptualization, Writing – original draft. YD: Conceptualization, Writing – review & editing. SL: Conceptualization, Writing – review & editing. Y-QG: Writing – review & editing. JL: Writing – review & editing.
